# Can heart rate variability data from the Apple Watch electrocardiogram quantify stress?

**DOI:** 10.3389/fpubh.2023.1178491

**Published:** 2023-07-05

**Authors:** Pedro Elkind Velmovitsky, Matheus Lotto, Paulo Alencar, Scott T. Leatherdale, Donald Cowan, Plinio Pelegrini Morita

**Affiliations:** ^1^School of Public Health Sciences, University of Waterloo, Waterloo, ON, Canada; ^2^Department of Pediatric Dentistry, Orthodontics and Public Health, Bauru School of Dentistry, University of São, Paulo, Bauru, Brazil; ^3^David R. Cheriton School of Computer Science, University of Waterloo, Waterloo, ON, Canada; ^4^Research Institute for Aging, University of Waterloo, Waterloo, ON, Canada; ^5^Department of Systems Design Engineering, University of Waterloo, Waterloo, ON, Canada; ^6^Institute of Health Policy, Management, and Evaluation, Dalla Lana School of Public Health, University of Toronto, Toronto, ON, Canada; ^7^Centre for Digital Therapeutics, Techna Institute, University Health Network, Toronto, ON, Canada

**Keywords:** mHealth, stress, heart rate, mobile, wearable, ECG, Apple Watch

## Abstract

Chronic stress has become an epidemic with negative health risks including cardiovascular disease, hypertension, and diabetes. Traditional methods of stress measurement and monitoring typically relies on self-reporting. However, wearable smart technologies offer a novel strategy to continuously and non-invasively collect objective health data in the real-world. A novel electrocardiogram (ECG) feature has recently been introduced to the Apple Watch device. Interestingly, ECG data can be used to derive Heart Rate Variability (HRV) features commonly used in the identification of stress, suggesting that the Apple Watch ECG app could potentially be utilized as a simple, cost-effective, and minimally invasive tool to monitor individual stress levels. Here we collected ECG data using the Apple Watch from 36 health participants during their daily routines. Heart rate variability (HRV) features from the ECG were extracted and analyzed against self-reported stress questionnaires based on the DASS-21 questionnaire and a single-item LIKERT-type scale. Repeated measures ANOVA tests did not find any statistical significance. Spearman correlation found very weak correlations (*p* < 0.05) between several HRV features and each questionnaire. The results indicate that the Apple Watch ECG cannot be used for quantifying stress with traditional statistical methods, although future directions of research (e.g., use of additional parameters and Machine Learning) could potentially improve stress quantification with the device.

## 1. Introduction

According to the WHO, stress is the “Health Epidemic of the 21st Century” (1). Over a quarter of U.S. adults report such high levels of daily stress that they are not able to function properly (2). Stress, as a survival mechanism, is normal and healthy: stress allows the body to generate more energy to deal with a potential threat. The stress response is modulated by the sympathetic nervous system (SNS) and parasympathetic nervous system (PNS). The SNS is responsible for triggering a response to unexpected threats to generate energy and resources for the body – the fight-or-flight response – by signalling adrenal glands to release adrenalin and cortisol, which lead to several physiological changes including increased heart rate, blood pressure, and respiration (3, 4). Once the acute stressors are removed, the PNS functions to relax the body, returning it to its normal state (3, 4).

Despite the necessity of a stress response to survival, chronic exposure to stressors can lead to severe health consequences including cardiovascular diseases, hypertension, obesity, and diabetes (3, 5, 6). Chronic stress is an increasingly observed condition worldwide. High levels of daily stress are reported by 38% of United States adults aged 40–49 years and 33% of adults aged 50–59 years (7). In Canada, daily stress was highest amongst individuals between 35–49 years (27.8%) followed by individuals aged 50–64 years (22%) and 18–34 years (21.9%) (8). Individuals over 65 years reported the lowest levels of stress (8). Chronic stress is estimated to cost over USD 300 billion annually in associated healthcare expenses, reduced job performance, and absenteeism (1, 9). Workplace stress is connected with 120,000 premature deaths annually (10). The COVID-19 pandemic has amplified this crisis: a recent survey by the American Psychological Association discovered that approximately 80% of respondents identify the pandemic as a major source of stress in their life and almost 70% reported increased levels of stress owing to COVID-19 (11).

The identification of stress and the application of interventions should be a public health priority. Research data on stress is typically collected through self-reporting surveys, which may have limitations such as low response rates, recall and social bias, cost and delays (12). Smart technologies, such as mobile and wearable devices, have recently been identified as useful tools to measure health parameters. Several of these technologies have embedded sensors that collect objective health data such as sleep, blood pressure, and heart rate (13, 14). In particular, an electrocardiogram (ECG) feature for detecting atrial fibrillation has been introduced to the Apple Watch device (13–15). Unlike the standard 12-lead ECGs, which use electrodes connected to the body, the Apple Watch ECG collects a 30-s 1-lead ECG when users place their finger on an electrode located in the digital crown of the device (16). Interestingly, ECG data can be used to derive Heart Rate Variability (HRV) features which are commonly used in the identification of stress (17). This suggests that the Apple Watch ECG app could potentially identify and monitor individual stress. Apple Watch applications could use this information to provide instant user feedback and interventions, such as suggesting the use of meditation apps (18). Furthermore, the use of a wearable data collection device would improve stress research data by eliminating recall biases and increasing population sample sizes. However, compared to longer measurements, there is not a large amount of evidence suggesting that ultra-short HRV measurements are reliable (19).

The goal of this paper was to explore the associations between HRV data collected from the Apple Watch ECG app with perceived stress levels in a real-life study. To the best of our knowledge, this is the first paper that provides statistical analyses of data derived from the Apple Watch ECG for stress detection, studying the reliability of these short-term measurements, and it is a continuation of previous work that uses a set of the same data, from 40 participants, to create Machine Learning (ML) stress prediction models (12). ECG data from the Apple Watch ECG app was collected from 36 participants in a real-world setting over 2 weeks. We were able to identify significant, albeit weak, correlations between several HRV features and self-reported stress states, as well as significant differences between groups. Results from this study support the continued development of wearable ECG sensors as tools to measure stress.

The paper is organized as follows: section 2 described related work, including previous studies that used different sets of the same data for creating Machine Learning models; section 3 describes the methods, while section 4 presents the results and section 5 discusses our findings. Finally, section 6 presents the conclusions.

## 2. Related work

This paper is an extension of previous work performed by the authors that uses data from 40 participants, to derive HRV features from the Apple Watch ECG data and use that data to create machine learning (ML) models for stress prediction – specifically using Random Forest and Support Vector Machines (12). The models, trained on subsets of the data according to age, gender, income, profession, and health status, found a weighted f1-score lying approximately between 55–65%, which is in line with the state-of-the-art for stress prediction using ML, although towards the low end. The models possessed high specificity – i.e., in general they were capable of successfully predicting when an individual is not stressed – but were less successful when predicting the stressed state. Notably, feature importance of the Random Forest models was calculated to determine, for each model, what features were most important in determining the prediction results. Although they vary per model, in general the heart’s acceleration (AC) and deceleration (DC) capacity were some of the most important features, present in most of the models. Another noteworthy feature is the standard deviation of interbeat intervals (SDNN). A more detailed explanation of HRV features and the feature extraction process is provided in the methods section.

Data from the same study, this time from 27 participants, was used by Benchekroun et al. (20), although in this case the HRV data was derived from the Empatica E4 device rather than from the Apple Watch ECG. The Empatica E4 device collects data continually as opposed to cross-sectionally, providing larger datasets. Random Forests trained on this data in an area under the receiver operating characteristic (ROC) curve (ROC AUC) of 0.79 and a macro f1-score of 75%. Further, a cross dataset analysis was performed in which models were trained on a laboratory dataset and tested on the Empatica E4 data, achieving a ROC AUC of 65% and a f1-macro score of 62%.

MCcraty et al. (21) performed repeated measures ANOVA analysis on HRV metrics of 24 patients with panic disorder and healthy control, finding differences in features such as the SDNN index, Total Power of VLF, Normalized LF/HF ratio, among others. Hong et al. (22) conducted repeated ANOVA analyses for participants, finding changes in HF and RMSDD.

Seipäjärvi et al. (23) studies stress and HRV in a laboratory setting among participants in different age groups and health status, finding that with the application of stressors differences in HRV could be observed. Föhr et al. (24) investigated the association between physical activity, HRV and subjective stress measured with the perceived stress scale (PSS), finding significant changes between physical activity and HRV with stress. However, using ecological momentary assessments, Martinez et al. (25) found a significant but small relationship between HRV and stress, where only a small amount of variance was explained by models. The author’s concluded that HRV might be a good proxy for stress in controlled settings with specific stressors applied, but not in real-life. Silva et al. (26) conducted Spearman correlation analysis between the perceived stress scale (PSS-14) and 5-min HRV variables at rest, and found weak to moderate correlation for the low frequency (LF) band. A similar Spearman correlation analysis was done in this study between HRV features and stress.

The Task Force of the European Society of Cardiology and the North American Society of Pacing and Electrophysiology provide widely used guidelines for the analyses of HRV data and were of great help in guiding this research (27). The authors in Acharya et al. provided an extensive review of HRV metrics (17), while several papers explored the feasibility and characteristics of analyzing HRV data. For example, Benchekroun et al. (28) discussed the impact of missing data on several HRV-related metrics and the best interpolation techniques to handle this situation.

It is important to note that there is limited research on the reliability of ultra-short-term HRV measurements (less than 5 min) when compared to long-term methods. Baek et al. studies ultra-short-term measurements to define recommended minimum intervals for each of these metrics to be valid (19). In general, each metric has a different recommended interval, varying from seconds to minutes. Shaffer et al. (29) conducted a review of ultra-short-term heart rate variability norms, finding that most studies did not use criterion validity to study if the procedures produce comparable results with validates measurement procedures, applying other metrics (e.g., Pearson correlation) which may be insufficient to provide evidence of comparable methods. Studies that did use more appropriate metrics [such as Baek et al. (19) mentioned previously] typically found that different metrics will depend on different intervals. Munoz et al. (30), for example, found that a minimum of 10s was required for RMSSD and 30s for SDNN. The authors also found that ultra-short-term measurements are extremely sensitive to artifacts. For example, a single false heartbeat can alter the HRV metrics, and so special care must be taken when analyzing the data. In short, while ultra-short-term recordings such as the ones used in this study have potential due to its increased accessibility and ease-of-use, there is a lack of robust evidence base to assert that these recordings can be used as proxies for longer recordings. In this study, as will be described, the Kubios Premium Sofware was used to process the data to mitigate issues with noise or artificats.

In addition, while Apple Watch ECG data was shown to be successful in detecting atrial fibrillation (31) there is also a lack of a robust evidence base on how the HRV data derived from the Apple Watch ECG compares to gold standards. A study by Saghir et al. (32) found good results, showing that the agreement between the Apple Watch ECG and a standard 12-lead ECGs to be moderate to strong in health adults. In other words, there is promising but limited evidence both on ultra-short-term recordings and on how Apple Watch ECG data compares to more traditional, longer-term measurement methods.

It should also be noted that, while on this work we are specifically focusing on HRV derived from ECG – HRV being an essential parameter in stress quantification – other metrics, such as electrodermal activity (EDA), can also be considered for analyses (33).

## 3. Methods

### 3.1. Participant recruitment

Healthy participants (*n* = 36) were recruited from the University of Waterloo as well as through Facebook Ads and Kijiji (a Canadian website that allows users to advertise products and services). Participants had to live close to the Kitchener-Waterloo region in Ontario for devices to be delivered in person. Participants were offered CAD 100.00 for 2 weeks of data collection. This study was approved by the University Waterloo Research Ethics Board (REB [43612]). Data collection took place between December 2021 and December 2022. Table 1 shows the characteristics of the study participants. Participants were aged 18 years or older. For the analyses described in this paper, we considered only healthy participants, i.e., who did not drink or smoke, did not have any chronic conditions or take prescription medications.

**Table 1:** Study population characteristics.

Participants (*N* = 36)	Frequency	Percentage
*Age*		
18–24	12	33
25–34	10	28
35–44	10	28
45–64	3	8
Above 65	1	3
*Gender*		
Male	13	36
Female	23	64
*SES*		
Low (0-$30,000)	16	44
Medium ($30,000– $100,000)	13	36
High (above $100,000)	4	12
Do not wish to disclose	3	8
*Profession*		
Full-time	17	47
Part-time	3	8
Student	13	36
Self-employed/other	2	6
Retired	1	3
*Ethnicity*		
Black or African American	3	8
Black and Southeast Asian	1	3
Chinese	4	11
Indian	1	3
Latin American	7	19
South Asian	11	31
White	9	25
*Self-reported stress level, beginning of study*		
1. Great	0	0
2. Good	8	22
3. A little stressed	15	42
4. Definitely stressed	11	31
5. Stressed out	2	6

### 3.2. Data collection

This study followed the Ecological Momentary Assessment (EMA) methodology to obtain self-reports closer to the event to approximate real-life scenarios (34). Participants were given an iPhone 7 with iOS 15.0 and an Apple Watch Series 6 with watch OS 8.3 for 2 weeks. The Apple Watch contained the ECG app and a Mobile Health Platform (MHP) was installed on the iPhone. The MHP was used to collect health data, including ECG recordings, from the iPhone’s Apple Health app data repository (12–14, 20).

**Figure 1:**
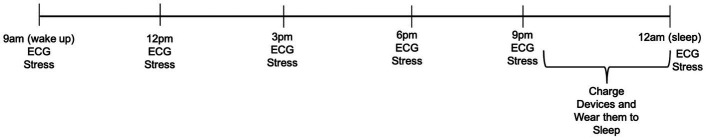
Study protocol.

Users were instructed to perform an ECG measurement on the Apple Watch ECG app 6 times during the day in approximately three-hour intervals followed by the stress questionnaire (below) on the iPhone. Figure 1 shows the study protocol (times are included for reference purposes; participants were asked to collect data as soon as they woke up).

**Figure 2:**
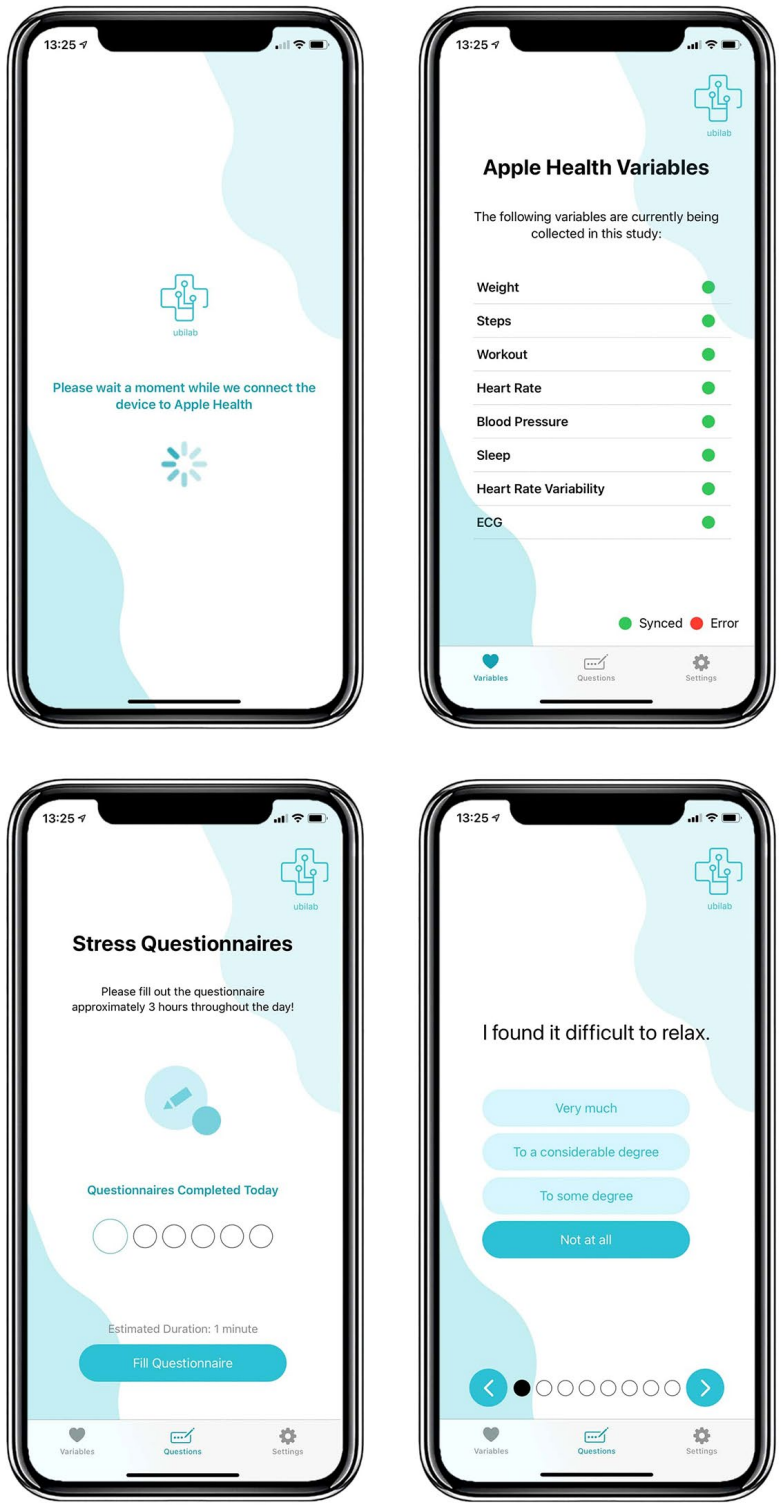
MHP interface.

The app installed in the iPhone, termed the Mobile Health Platform (MHP), can collect health data saved on the iPhone’s health data repository, the Apple Health app, including the ECG recordings. The MHP collected this data, which were then saved in our database using the JSON format (for each ECG reading there are 15,360 voltage measurements and associated timestamps in milliseconds, forming the 30-s ECG). The MHP also contains a tab with the stress questionnaires to be completed, which will be described next. Figure 2 shows the interface of the MHP, including the additional variables collected in the study.

We noticed that several participants had difficulty managing the study protocol with their daily life responsibilities. Therefore, we asked participants to use the devices for additional days to compensate as applicable.

Of note, this study is part of a larger cross-sectional study that investigates the use of smart technologies for stress detection. As part of this larger study, in addition to the Apple Watch and iPhone, participants were also given additional devices capable of collecting other data, such as the Withings Blood Pressure Monitor and the Empatica E4. Since this is not the focus of the paper we will not describe the use of these devices further, but more information on these expanded protocols is provided in Velmovitsky et al. (12–14) and Benchekroun et al. (20).

### 3.3. Stress questionnaires

As there are a limited number of validated stress questionnaires for the EMA with a validation period relevant to this study, we used the stress subscale of the Depression, Anxiety, and Stress Scale (DASS-21) for our stress questionnaire. While the DASS-21 is usually applied over a week, there is promising evidence of using DASS-21 with EMA (35). In addition, Wang et al. (36) used a single-item measure that, while lacking validation in the literature, was used successfully for stress prediction and is moderately correlated with robust stress questionnaires. The following questionnaire on a LIKERT-type scale was used for our study. Questions 1–7 are related to the DASS-21 and question 8 comprises the single-item measure used by Wang et al.

1. I found it hard to wind down
2. I felt that I was using a lot of nervous energy
3. I found myself getting agitated
4. I found it difficult to relax
5. I tended to over-react to situations
6. I was intolerant of anything that kept me from getting on with what I was doing
7. I felt that I was rather touchy
8. Right now, I am…

Questions 1–7 have the options: “Not at all,” “To some degree,” “To a considerable degree,” and “Very much,” while Question 8 has “Stressed Out,” “Definitely stressed,” “A little stressed,” “Feeling good,” and “Feeling great.” The questions were displayed to the user in a random order each time the questionnaire was filled in the MHP, and compose the perceived stress, i.e., the degree to which a stressfull situation affects an individual, is measured.

In addition to self-reporting stress throughout the day, participants were asked to self-report their stress levels at the beginning of the study with the single-item measure (results shown in Table 1).

### 3.4. Data pre-processing

To obtain the HRV features from the ECG readings, we made use of Kubios Premium 3.5.0, a widely used software that analyzes and extracts features from several heart-related signals (17, 37). The JSON ECG data was exported into a CSV format and each voltage measurement was sorted by timestamp. The CSV file was imported into Kubios.

Kubios automatic beat correction feature was used and any samples that contained more than 5% of corrected beats were removed. In addition, any ECG sample classified as Poor Recording or Inconclusive by the ECG app was also removed from the analysis (16). Frequency features were calculated using both the Fast Fourier Transform (FFT) and Autoregressive Spectral Analysis (AR). A list of the features generated by Kubios based on the 30-s ECG signal is presented in Table 2 (17, 37).

**Table 2:** Kubios HRV features derived from Apple Watch ECG.

Name	Description
ECG_Mean HR	Mean of heart rate from ECG(ms)
ECG_ SD HR	Standard deviation of instantaneous heart rate from ECG (1/min)
ECG_Min HR	Minimum instantaneous heart rate calculated using 5 beat moving average from ECG(1/min)
ECG_Max HR	Maximum instantaneous heart rate calculated using 5 beat moving average from ECG (1/min)
HRV-1	Heart rate variability collected as SDNN with the Apple Watch
ECG_PNS Index	Parasympathetic nervous system activity compared to normal resting values
ECG_SNS Index	Sympathetic nervous system activity compared to normal resting values
ECG_Stress Index	Square root of Baevsky’s stress index
ECG_Mean RR	Mean of R-R intervals (ms)
ECG_SDNN	Standard deviation of R-R intervals (ms)
ECG_RMSSD	Square root of the mean squared differences between successive RR intervals f(ms)
ECG_DC	Heart rate deceleration capacity (ms)
ECG_DCMod	Modified DC computer as a two-point difference (ms)
ECG_AC	Heart rate acceleration capacity (ms)
ECG_ACMod	Modified AC computer as a two-point difference (ms)
ECG_FFT LF	Fast Fourier Transform Low Frequency band components (Hz)
ECG_FFT HF	Fast Fourier Transform High Frequency band components (Hz)
ECG_AR LF	Autoregressive Low Frequency band components (Hz)
ECG_AR HF	Autoregressive High Frequency band components (Hz)
ECG_FFT Absolute Power LF	Fast Fourier Transform Absolute Power of Low Frequency band components (ms2)
ECG_FFT Absolute Power HF	Fast Fourier Transform Absolute Power of High Frequency band components (ms2)
ECG_AR Absolute Power LF	Autoregressive Absolute Power of Low Frequency band components (ms2)
ECG_AR Absolute Power HF	Autoregressive Absolute Power of High Frequency band components (ms2)
ECG_FFT Relative Power LF	Fast Fourier Transform Relative Power of Low Frequency band components (%)
ECG_FFT Relative Power HF	Fast Fourier Transform Relative Power of High Frequency band components (%)
ECG_AR Relative Power LF	Autoregressive Relative Power of Low Frequency band components (%)
ECG_AR Relative Power HF	Autoregressive Relative Power of High Frequency band components (%)
ECG_FFT Normalized Power LF	Fast Fourier Transform Normalized Power of Low Frequency band components (n.u)
ECG_FFT Normalized Power HF	Fast Fourier Transform Normalized Power of High Frequency band components (n.u)
ECG_FFT Total Power	Fast Fourier Transform Total Power (ms2)
ECG_FFT LF/HF	Fast Fourier Transform ratio between low and high frequency
ECG_AR Normalized Power LF	Autoregressive Normalized Power of Low Frequency band components (n.u)
ECG_AR Normalized Power HF	Autoregressive Normalized Power of High Frequency band components (n.u)
ECG_AR Total Power	Autoregressive Total Power (ms2)
ECG_AR LF/HF	Autoregressive ratio between low and high frequency
ECG_SD1	The standard deviation perpendicular to the line-of-identity in Poincaré plot (ms)
ECG_SD2	The standard deviation along the line-of-identity in Poincaré plot (ms)
ECG_SD2/SD1	Ratio between SD2 and SD1 (ms)

The scores of the DASS-21 questions summed together were multiplied by 2. If the score was bigger than 14, the sample was classified as “stress” according to DASS-21 guidelines (38). For the single-item measure, the sample was classified as “stress” if the score was bigger than 2, as that would represent the user being at least “a little stressed.” If the DASS-21 score or the single-item score were classified as “stress,” the measurement was classified as the “stress” state.

### 3.5. Statistical analysis

Statistical analyses were performed through the Statistical Package for Social Sciences (v. 28.0; SPSS, Chicago, IL, United States). Using baseline stress scores from the Single-Item measure at the beginning of the study, repeated measures ANOVA analyses were conducted followed by Tukey’s Post-Hoc test in case of statistically significant features. In addition, Spearman’s non-parametric correlation test was applied to detect the correlation between each ECG variable with the quantitative DASS-21 and single Item questionnaire scores. For all analyses, *p* < 0.05 was considered statistically significant. While correlations were performed for every feature, to limit the potential of biases ANOVA analyses were conducted with a subset of the features (Table 3) as seen in other works (21, 22). In addition, for the analyses, we considered 13 days of data for each participant (the minimum days of all participants in the study).

**Table 3:** Repeated measures ANOVA for HRV parameters with baseline self-perceived stress (*p* < 0.05).

Parameter	Source	Sum of squares	Mean square	*F*	*p*-value
AC	Days	1,293	108	0.656	0.794
	Days × self-perceived stress	1,332	111	0.675	0.775
DC	Days	2,119	177	0.733	0.719
	Days × self-perceived stress	1,242	103	0.430	0.952
RMSSD	Days	1,390	116	0.443	0.945
	Days × self-perceived stress	2,293	191	0.731	0.721
SDNN	Days	678	56.5	0.372	0.973
	Days × self-perceived stress	667	55.6	0.366	0.975
Stress Index	Days	150	12.5	0.856	0.592
	Days × self-perceived stress	158	13.2	0.901	0.546
FFT Absolute Power LF	Days	8.52e+6	710,175	0.672	0.779
	Days × self-perceived stress	1.70e+7	1.41e+6	1.337	0.195
FFT Absolute Power HF	Days	8.85e+6	737,302	0.600	0.843
	Days × self-perceived stress	1.44e+7	1.20e+6	0.978	0.469
AR Absolute Power LF	Days	3.24e+7	2.70e+6	0.787	0.664
	Days × self-perceived stress	5.12e+7	4.27e+6	1.245	0.250
AR Absolute Power HF	Days	1.08e+9	8.96e+7	0.956	0.491
	Days × self-perceived stress	1.43e+9	1.19e+8	1.269	0.234
FFT Relative Power LF	Days	1,591	132.6	0.895	0.552
	Days × self-perceived stress	979	81.6	0.551	0.881
FFT Relative Power HF	Days	2048	171	1.031	0.419
	Days × self-perceived stress	1812	151	0.912	0.535
AR Relative Power LF	Days	2,136	178	1.091	0.366
	Days × self-perceived stress	1,431	119	0.731	0.721
AR Relative Power HF	Days	2,258	188	1.056	0.396
	Days × self-perceived stress	1829	152	0.856	0.593

## 4. Results

To determine whether HRV data collected from an Apple Watch ECG was associated with perceived stress level, we recruited 36 healthy participants to participate in a real-life study. Using the Apple Watch ECG app and an iPhone app developed for this study, users were instructed to collect ECG readings and complete a stress questionnaire 6 times during the day in approximately three-hour intervals for 2 weeks, as well as fill an initial survey about perceived stress levels prior to data collection. Table 2 lists the HRV features captured by the Apple Watch ECG. Questionnaires comprised 8 questions based on the DASS-21 (38) and the measure used by Wang et al. (36) as mentioned in the previous section.

Participants were predominantly female (64%; Table 1). 61% were employed and 36% were students. Participants were mostly South Asian, White, or Latin American (31, 25, and 19% respectively), and reported low to medium income (44 and 36%, respectively) the average of days a participant had in the study was 17.1 (±2.5), and an average of 59 (±16.0) ECG recordings. Participants were also asked to self-report their stress levels at the beginning of the study with the single-item measure (results shown in Table 1).

As described in the previous section, using the questionnaire score, measurements were designated as self-perceived “stress” if (a) the DASS-21 questions were classified as “stress” according to a DASS-21 greater than 14; or (b) the single-item measure was classified as “stress” if the score was greater than 2. Measurements that did not meet this cut-off were designated as “no stress.”

Repeated measures ANOVA test was performed to compare differences recorded by the Apple Watch ECG and self-perceived stress. No statistical significance was revealed (Table 3).

To determine which ECG variables correlated with stress, we applied a Spearman’s non-parametric correlation analysis between HRV features and self-perceived stress, divided by each of the stress scores (DASS-21 and Single-Item measure). Spearman correlation coefficients (*r*) and *p*-values were calculated and shown in Table 4.

**Table 4:** Correlation coefficients (r) and *p* value for Spearman’s non-parametric correlation analysis.

Variables	DASS-21		Single item	
	*r*	*p*	*r*	*p*
ECG PSN Index	0.039	0.070	−0.010	0.653
ECG SNS Index	−0.075	0.001*	−0.026	0.227
ECG Stress Index	−0.105	0.001*	−0.046	0.036*
ECG Mean RR	0.033	0.131	0.014	0.528
ECG SDNN	0.109	0.001*	0.044	0.041*
ECG Mean HR	−0.033	0.131	−0.014	0.528
ECG SD HR	0.102	0.001*	0.048	0.027*
ECG Min HR	−0.050	0.021*	−0.022	0.303
ECG Max HR	−0.014	0.522	−0.013	0.554
ECG RMSSD	0.077	0.001*	0.001	0.957
ECG NN50	0.064	0.003*	−0.003	0.892
ECG pNN50	0.069	0.001*	−0.002	0.925
ECG RR Tri Index	0.091	0.001*	0.034	0.120
ECG TINN	0.110	0.001*	0.045	0.038*
ECG DC	0.099	0.001*	0.058	0.008*
ECG Dcmod	0.076	0.001*	0.008	0.721
ECG AC	−0.105	0.001*	−0.073	0.001*
ECG ACmod	−0.075	0.001*	−0.014	0.528
ECG FFT VLF	−0.016	0.449	0.003	0.872
ECG FFT LF	0.042	0.051	0.014	0.528
ECG FFT HF	−0.061	0.005	−0.072	0.001*
ECG AR VLF	0.019	0.383	0.027	0.208
ECG AR LF	−0.003	0.875	−0.039	0.071
ECG AR HF	−0.082	0.001*	−0.084	0.001*
ECG FFT Absolute Power VLF	0.081	0.001*	0.051	0.020*
ECG FFT Absolute Power LF	0.102	0.001*	0.047	0.030*
ECG FFT Absolute Power HF	0.099	0.001*	0.033	0.127
ECG FFT Absolute Power VFL log	0.081	0.001*	0.051	0.020*
ECG FFT Absolute Power LF log	0.102	0.001*	0.047	0.030*
ECG FFT Absolute Power HF log	0.099	0.001*	0.033	0.127
ECG AR Absolute Power VLF	0.088	0.001*	0.032	0.134
ECG AR Absolute Power LF	0.099	0.001*	0.044	0.041*
ECG AR Absolute Power HF	0.085	0.001*	0.016	0.472
ECG AR Absolute Power VLF log	0.088	0.001*	0.032	0.134
ECG AR Absolute Power LF log	0.099	0.001*	0.044	0.041*
ECG AR Absolute Power HF log	0.085	0.001*	0.016	0.472
ECG FFT Relative Power VLF	−0.028	0.191	−0.003	0.903
ECG FFT Relative Power LF	−0.009	0.685	0.010	0.658
ECG FFT Relative Power HF	0.025	0.247	−0.006	0.782
ECG AR Relative Power VLF	−0.019	0.371	−0.009	0.684
ECG AR Relative Power LF	−0.009	0.676	0.032	0.135
ECG AR Relative Power HF	0.022	0.304	−0.020	0.350
ECG FFT Normalized Powers LF	−0.023	0.297	0.007	0.763
ECG FFT Normalized Powers HF	0.023	0.291	−0.006	0.788
ECG FFT Total Powers	0.110	0.001*	0.053	0.014*
ECG FFT LFHF	−0.023	0.292	0.006	0.773
ECG AR Normalized Powers LF	−0.019	0.393	0.025	0.254
ECG AR Normalized Powers HF	0.019	0.379	−0.024	0.267
ECG AR Total Power	0.099	0.001*	0.034	0.118
ECG AR LFHF	−0.019	0.385	0.024	0.262
ECG SD1	0.077	0.001*	0.001	0.953
ECG SD2	0.115	0.001*	0.057	0.008*
ECG SD1SD2	0.021	0.330	0.081	0.001*

Regarding DASS-21, several features were shown to have a weak correlation including: SNS Index, Stress Index, SDNN, SD HR, Min HR, RMSSD, NN50, pNN50, RR Tri Index, TINN, DC, DC mod, AC, AC mod, FFT Absolute Power VLF, FFT Absolute Power LF, FFT Absolute Power HF, FFT Absolute Power VLF log, FFT Absolute Power LF log, FFT Absolute Power HF log, AR Absolute Power VLF, AR Absolute Power LF, AR Absolute Power HF, AR Absolute Power VLF log, AR Absolute Power LF log, AR Absolute Power HF log, FFT Total Power, AR Total Power, SD1, SD2.

The Single-Item measure significant correlations were: Stress Index, SDNN, SD HR, TINN, DC, AC, FFT HF, FFT Absolute Power VLF, FFT Absolute Power LF, FFT Absolute Power VLF log, FFT Absolute Power LF log, AR Absolute Power LF, AR Absolute Power LF log, FFT Total Power, SD2, SD1/SD2.

## 5. Discussion

Overall, some HRV features captured by the Apple Watch weakly correlate to the stress questionnaires. Repeated measures ANOVA test and Tukey’s Post-Hoc test indicated that Apple Watch ECG features in the current study design cannot statistically differentiate between stress states in a real-world setting. Therefore, the answer of “can Heart Rate Variability data from the Apple Watch ECG Quantify Stress” with the use of the statistical methods investigated in this work seems to be no.

Regarding Spearman correlations, while several features in the domains (time domain, frequency domain, non-linear) were shown to have a significant correlation with the DASS-21 and single-item measure, all were weak. Nevertheless, interesting points can be made by comparing the differences between the two questionnaires.

In general, the significant correlations between HRV features and the single-item measure are a subset of the ones from DASS-21. One of the main differences in the correlations between the DASS-21 and the single-item measure is that the latter does not seem to be significantly correlated to the absolute power high frequency components (FFT Absolute Power HF and AR Absolute Power HF). In this way, the use of both questionnaires for the study seem to complement each other in capturing differing dimensions of self-perceived stress, although it should be noted that the weak correlations may limit the validity of these results.

Interestingly, Silva et al. (26), also found weak to moderate correlations using the Spearman test while comparing HRV metrics with stress from the PSS-14 questionnaire but failed to find any significant correlations except for the LF band. Given that participants’ measurements were taken at rest and the PSS-14 stress scores were in the mid to low range, it is possible that physiological changes owing to stress affected the correlation values in our current work.

Indeed, several factors may have affected the quality of the data. First, being a “real-life” experiment, data may be subjected to noise and errors in measurements. For example, respondents may forget to take measurements throughout the day, take the measurements incorrectly, or be influenced by the Hawthorne Effect in which respondents change their behaviour because they are being monitored. On the same token, elements such as sweat, or movement may affect the measurement. These factors may have influenced the results, leading to potentially inaccurate data. Future work should explore data collection of ECG in controlled conditions, potentially with an intervention (e.g., applying stressors in a lab) to evaluate the robustness of this data. This recommendation is also in line with Martinez et al. conclusions that HRV may be best represented in controlled environments with specific stressors (25). While this would diminish the validity of ECG data to be used in real-life scenarios to identify stress, it could provide further clues as to how the relationship between these variables work and new directions of research. Further, future work on this dataset can consider the distribution of the data per day and HRV diurnal fluctuations, which could provide more significant and illuminating results.

In addition, a convenience sample was used in this pilot study, and as can be seen by Table 2, there is a predominance of females and participants with low to medium SES which may affect the external validity of the results. Finally, since we used the EMA methodology, we decided to combine both the DASS-21 and the single-item measure for stress classification, which can potentially affect how individuals report stress and may lead to some of the contradictory findings in terms of group differences presented here. On that note, this study focused on perceived stress, i.e., the degree to which a situation perceived as stressful affects individuals. In this context, subjective ratings of stress may be affected by each participant’s internalized definition of stress, which in turn may influence responses (39). Nevertheless, the fact that several significant – albeit weak – correlations were found are encouraging and additional, more controlled, and stratified experiments should be conducted to confirm and clarify these relationships between the HRV features from the Apple Watch ECG and self-perceived stress.

As described in the Related Work section, there is promising but limited evidence on the reliability of ultra-short-term measurements and the Apple Watch ECG when compared to traditional measurement methods and data. It is possible that inaccuracies in the Apple Watch ECG led to a lack of statistical differences between stress states in this study. In addition to controlled experiments, future research could also consider using different methods of ultra-short-term data collection to verify the results. Given that weak correlations were found, the use of additional parameters in addition to simply the Apple Watch ECG might also help with quantifying stress. Indeed, several physiological and behavioural variables have been widely used in stress research. This could include brain activity measured through electroencephalogram (EEG), electrodermal activity (EDA), speech, mobile phone usage, among others (5). Physical activity (24, 40, 41) and sleep (40, 42, 43) could also be potentially used to discriminate stress and can also be collected passively with the Apple Watch sensors – if ECG and other Apple Watch data were successfully used in conjunction to differentiate between stressed states, potential solutions could focus simply on the Apple Watch for stress quantification, which would be of great value in studying the prevalence of these conditions and providing feedback to users. Finally, the use of Machine Learning for prediction, as previously mentioned, has shown promising results (12), and further studies also using other parameters could help improve prediction accuracy and realize the potential of the Apple Watch for stress studies.

## 6. Conclusion

The use of an Apple Watch ECG to quantify individual stress was piloted in a real-world scenario. Significant but weak correlations were found between several HRV features and measures of self-perceived stress. This study highlights the potential usefulness of the Apple Watch ECG as a minimally invasive tool for stress monitoring, quantification, and intervention, although more robust evidence is needed to establish the relationships between the data and its relevancy.

## Data availability statement

The original contributions presented in the study are included in the article/supplementary materials, further inquiries can be directed to the corresponding author: plinio.morita@uwaterloo.ca.

## Ethics statement

The studies involving human participants were reviewed and approved by University Waterloo Research Ethics Board (REB [43612]). The patients/participants provided their written informed consent to participate in this study.

## Author contributions

PV was responsible for conducting the study, collecting, and analysing the data, and writing the manuscript. ML provided help in the statistical analyses and in the writing of the manuscript. PM, PA, SL, and DC provided direction to the manuscript’s writing and preparation as well as editing and revision. All authors contributed to the article and approved the submitted version.

## Funding

This work was supported by an Ontario Trillium Scholarship from the Ontario Government.

## Conflict of interest

The authors declare that the research was conducted in the absence of any commercial or financial relationships that could be construed as a potential conflict of interest.

## Publisher’s note

All claims expressed in this article are solely those of the authors and do not necessarily represent those of their affiliated organizations, or those of the publisher, the editors and the reviewers. Any product that may be evaluated in this article, or claim that may be made by its manufacturer, is not guaranteed or endorsed by the publisher.
